# Characterization, Antioxidant Capacity and Protective Effect of Peptides from *Cordyceps militaris* Cultivated with Tussah Pupa on Oxidative Injured HepG2 Cells

**DOI:** 10.4014/jmb.2312.12012

**Published:** 2024-04-18

**Authors:** Bingxin Li, Jinying Zhang, Yefei Liu, Ze Wang, Fangxu Xu

**Affiliations:** 1 College of Life Science, Shenyang Normal University, Shenyang, Liaoning 110034, P.R. China; 2 Experimental Teaching Center, Shenyang Normal University, Shenyang, Liaoning 110034, P.R. China; 3 Key Laboratory of *Cordyceps militaris* with Functional Value of Liaoning Province, Shenyang, Liaoning 110034, P.R. China; 4 Industrial Technology Research Academy for *Cordyceps militaris* with Functional Value of Shenyang, Shenyang, Liaoning 110034, P.R. China; 5 *Cordyceps militaris* Germplasm Bank of Liaoning Province, Shenyang, Liaoning 110034, P.R. China

**Keywords:** Peptides, *Cordyceps militaris*, HepG2 cells, antioxidant capacity, protective effect

## Abstract

The antioxidant capacity and protective effect of peptides from protein hydrolysate of *Cordyceps militaris* cultivated with tussah pupa (ECPs) on H_2_O_2_-injured HepG2 cells were studied. Results indicated ECP1 (<3 kDa) presented the strongest antioxidant activity compared with other molecular weight peptides. Pretreated with ECPs observably enhanced survival rates and reduced apoptosis rates of HepG2 cells. ECPs treatment decreased the ROS level, MDA content and increased CAT and GSH-Px activities of HepG2 cells. Besides, the morphologies of natural peptides from *C. militaris* cultivated with tussah pupa (NCP1) and ECP1 were observed by scanning electron microscopy (SEM). Characterization results suggested the structure of NCP1 was changed by enzymatic hydrolysis treatment. Most of hydrophobic and acidic amino acids contents (ACC) in ECP1 were also observably improved by enzymatic hydrolysis. In conclusion, low molecular weight peptides had potential value in the development of cosmetics and health food.

## Introduction

Reactive oxygen species (ROS) mainly included free radicals and peroxides, etc., which have important biological functions in organisms [1, 2]. Oxidative stress refers to a physiological phenomenon in which the imbalance of oxidative and antioxidant processes in organisms causes the production of excessive ROS, which results into cell damage and disease [3]. It has reported that the occurrence of some chronic diseases is closely related to excess free radicals in the body, mainly including hydroxyl radical (HO^·^), superoxide anion radical (O_2_^-·^) and DPPH radical (DPPH^·^) [4]. Under oxidative stress, excessive ROS will not only change cell signal transduction pathway, causing protein and DNA damage, but also oxidize polyunsaturated fatty acids and destroy cell membrane structure, resulting in cell damage and apoptosis [5, 6].

As a kind of bioactive substance, peptides have the pharmacological effects of anti-oxidation [7], anti-inflammatory [8], anti-aging [9], hypoglycemic [10, 11], antibacterial [12] and anti-tumor [13], which play an indispensable role in cell physiological regulation, metabolic regulation and biological information transmission [14]. *C. militaris* cultivated with tussah pupa has attracted much attention because it is rich in a large number of functional components, particularly protein [15-16]. Therefore, *C. militaris* cultivated with tussah pupa is excellent raw material for preparing bioactive peptides.

Up to now, the development of peptides products has become an important direction of deep processing of *C. militaris* cultivated with tussah pupa [17-20]. However, compared with the strong industrialization demand of peptides products, the corresponding basic research on antioxidant function has not been systematically carried out. Our previous studies proved that peptides from protein hydrolysate had stronger antioxidant capacity than natural peptides from *C. militaris* cultivated with tussah pupa (NCP1) [21]. But the characterization, antioxidant capacity and protective effect of peptides from protein hydrolysate on oxidative injured HepG2 cells has not been thoroughly studied. Therefore, the enzymatic hydrolysis peptides from *C. militaris* cultivated with tussah pupa (ECPs) were prepared, and the antioxidant capacity of ECPs were evaluated by determining T-AOC, HO^·^, O_2_^-·^ and DPPH^·^ scavenging capacity. Besides, due to the fast growth rate, short culture cycle, simple culture conditions and high survival rate, HepG2 cells are continually applied as cell reactors to build different oxidative stress models for the screening of antioxidants. So, the protection of ECPs on H_2_O_2_-mediated oxidative injured HepG2 cells was investigated. Finally, scanning electron microscopy (SEM) observation, differential scanning calorimetry (DSC), fourier transform infrared spectroscopy (FT-IR), x-ray diffractometry (XRD) and amino acid contents (AAC) between NCP1 and ECP1 were conducted. The purpose of the experiment was to offer reference for the application of ECPs in the research and development of cosmetics and health food.

## Material and Methods

### Materials

*Cordyceps militaris* cultivated with tussah pupa were cultivated by Key Laboratory of *C. militaris* with Functional Value of Liaoning Province. HepG2 cells were bought from American Type Culture Collection (ATCC). Antioxidant capacity assay kits were bought from Nanjing Jiancheng Biotechnology Co., Ltd in China. ROS level, MDA content, CAT and GSH-Px activities assay kits were bought from Merck in China.

### Preparation of ECPs

The preparation of protein in *C. militaris* cultivated with tussah pupa was conducted in accordance with the method of Guo *et al*. [15]. Dried powder (100 g) of *C. militaris* cultivated with tussah pupa was added with 2,000 ml of deionized water, and then soaked for 0.5 h. Afterwards, the solution was titrated with 0.1 M NaOH and the pH value was adjusted to 8.0. Then the solution was treated three times with ultrasonic bath (100 W) and thermal reflux extraction device at 50°C for 0.5 h. The extracting solution were combined, whereafter titrated with 0.5 M HCl and the pH value was adjusted to 5.0. The solution was centrifuged at 6,000 × g for 5 min and the supernatant was used for the extraction of peptides. Alcalase and trypsin was used to hydrolyze the protein of *C. militaris* cultivated with tussah pupa with the following conditions: the ratio of alcalase and trypsin, 4:3; enzyme dosage, 7000 U mL^−1^; temperature, 55°C; pH, 7.2; hydrolysis time, 3.5 h. The hydrolysate was centrifuged at 6,000 × g for 10 min, and then the supernatant was lyophilized after being ultrafiltered into four fractions of different molecular weight, named ECP1 (< 3 kDa), ECP2 (3–10 kDa), ECP3 (10–30 kDa) and ECP4 (30–100 kDa), respectively.

### Determination of Antioxidant Capacity of ECPs

Total antioxidant capacity (T-AOC), hydroxyl radical (HO^·^), superoxide anion radical (O_2_^-·^) and DPPH radical (DPPH^·^) scavenging capacity of ECPs in *C. militaris* cultivated with tussah pupa at different concentrations were measured in accordance with the specifications of assay kits.

### HepG2 Cell Preparation

Cells were cultured for 24 h according to conventional methods [3]. Whereafter, cells were divided into the following groups. (1) Control group: 100 μl of culture medium were added, then incubated for 24 h. (2) ECPs protective group: 100 μl of 1, 1.5, 2.3, 3.4, 5.1 and 7.6 mg ml^–1^ ECPs (dissolved with DMEM) were added, then incubated for 24 h. Whereafter, 100 μl of H_2_O_2_ (800 μmol l^–1^) were added and incubated for 2 h. (3) Oxidation damage group: 100 μl of DMEM medium were added, then incubated for 24 h. Whereafter, 100 μl of H_2_O_2_ (800 μmol l^–1^) were added and incubated for 2 h. (4) Blank group: ECPs were added without HepG2 cells, then incubated for 24 h.

### Determination of Survival Rate and Apoptosis Rate

The determination of survival rate and apoptosis rate was conducted in line with the conventional instructions of Zhuang *et al*. [22]. The absorbance at 490 nm was measured and the calculation formula was as follows: survival rate (%) = (OD_experimental group_ - OD_blank group_)/(OD_control group_ - OD_blank group_) × 100%. The apoptosis rate was measured by a flow cytometer (FACSCanto II, USA) in accordance with the rules of Annexin V-FITC kit.

### Measurement of ROS Level and MDA Content in HepG2 Cells

Cells of the groups were collected and centrifuged for 10 min at 2,000 ×*g*. Cell deposits were obtained and treated on the basis of the instructions of assay kits.

### Measurement of CAT and GSH-Px Activities in HepG2 Cells

Cell deposits were obtained and the measurement of enzyme activity was conducted on the basis of the instructions of CAT and GSH-Px assay kits.

### SEM

The micromorphology of NCP1 and ECP1 were observed by a scanning electron microscope (460L, Thermo Fisher Scientific, USA). Samples were fixed on the platform after gold spraying, and the surface morphologies of NCP1 and ECP1 were observed and photographed, respectively.

### DSC

The thermograms of samples were recorded by a circular dichroism spectrometer (J-810, JASCO, Japan). Samples (0.25 mg) were sealed in the aluminum case, then placed on the support of circular dichroism spectrometer. A sealed aluminum case was applied for control. The determination conditions were as follows: heating rate, 10°C min^–1^; nitrogen pressure, 0.05 MPa; temperature range, 20–130°C.

### FT-IR

Samples were mixed and tableted to a film for analysis by an infrared spectrometer (Spectrum Two, USA) with the spectrum range of 4,000–500 cm^–1^.

### X-Ray Diffractometry (XRD)

The samples were fixed to the sample plate for scan using a x-ray powder diffractometer (SmartLab SE, Japan). The determination conditions were as follows: scanning range 2θ, 0–90°; scanning speed, 4° min^–1^; tube pressure, 40 kV; tube flow, 10 mA.

### Amino Acid Contents (AAC)

The samples were placed in hydrolysate tubes respectively and hydrolysed with 5 mol l^–1^ HCl solution at 120°C for 20 h. Then the concentration was diluted to 10-50 mg l^–1^ with salicylic acid after the hydrolysate dried. Then the derivatization was conducted. Finally, 50 μl of sample was taken and analyzed for amino acid composition and contents by an amino acid analyzer (L-8900, Hitachi, Japan).

### Statistical Analysis

Data were processed by the software of Statistic Package for Social Science 21.0. Significant differences were analysed with Duncan’s multiple range tests at 5% level. Data were exhibited as mean ± SE. All experiments were performed in triplicate.

## Results and Discussion

### Antioxidant Capacity of ECPs

Free radicals in the body are in a balanced state under normal circumstances. Once the balance is broken, it will not only cause damage to DNA and cell membranes, but also attack protein molecules, which resulting in the break of peptide chains, the generation of polymers and the oxidation of side chain amino acids [1]. Eventually, the function of protein is lost, thereby causing irreversible damage.

**Fig. 1:**
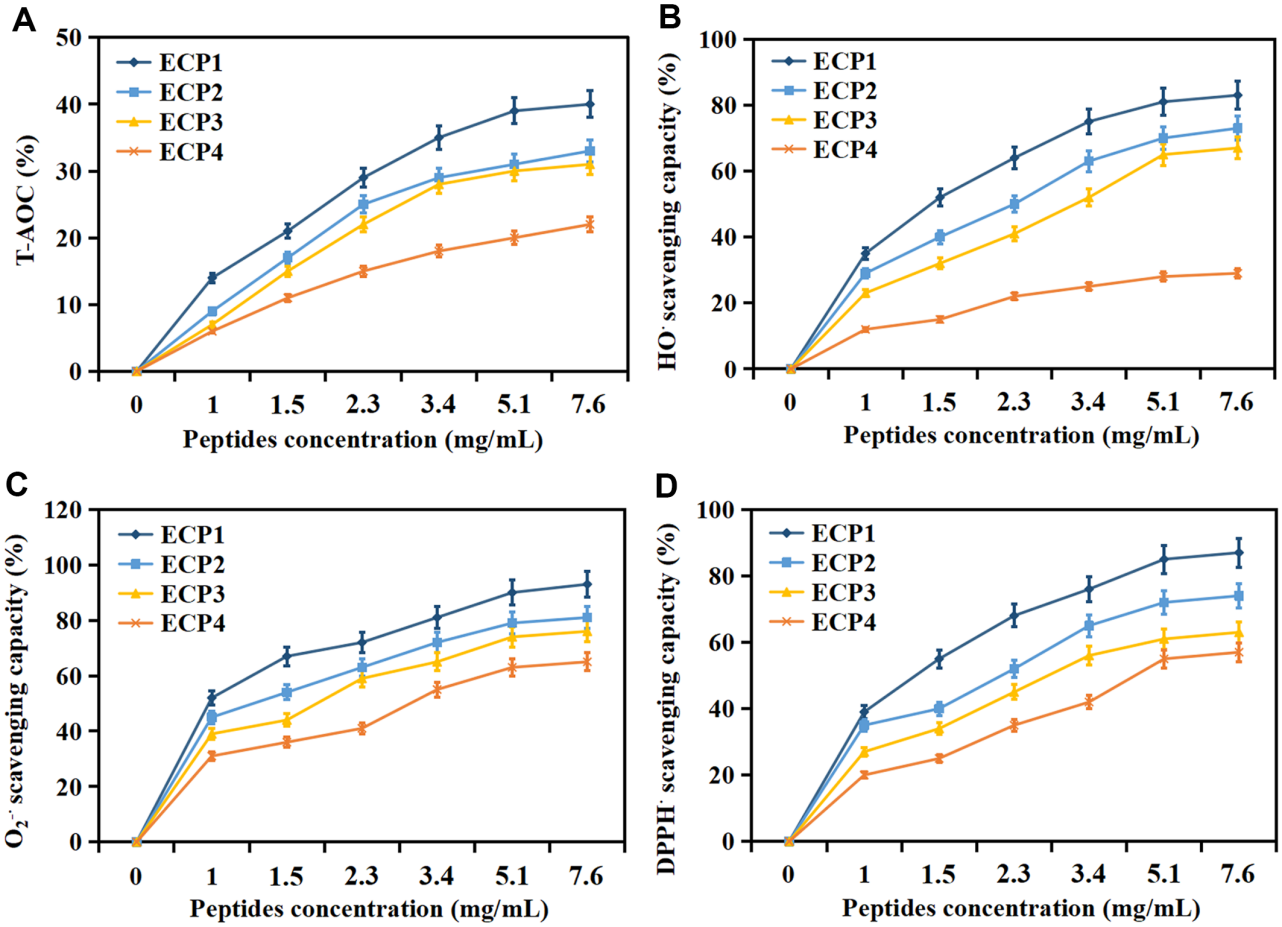
T-AOC (A), HO^·^ (B), O_2_^-·^ (C) and DPPH^·^ (D) scavenging capacity of ECPs at different concentrations. Values are mean of triplicate samples.

The antioxidant capacity of ECPs at different concentrations were evaluated by T-AOC, HO^·^, O_2_^-·^ and DPPH^·^ scavenging capacity (Fig. 1A). The radical scavenging capacity of ECPs showed an uptrend with the increase of peptide concentration. Notably, ECP1 exhibited the strongest antioxidant capacity compared to other molecular weight peptides. The results indicated that peptides with molecular weight less than 3 kDa had the best antioxidant effect, indicating its potential value in the development of cosmetics and health food. Analogous results were found by Guo *et al*. [15], who also considered peptides in *C. militaris* cultivated with tussah pupa showed a remarkable scavenging effect on DPPH and HO free radical.

### Survival Rate

**Fig. 2:**
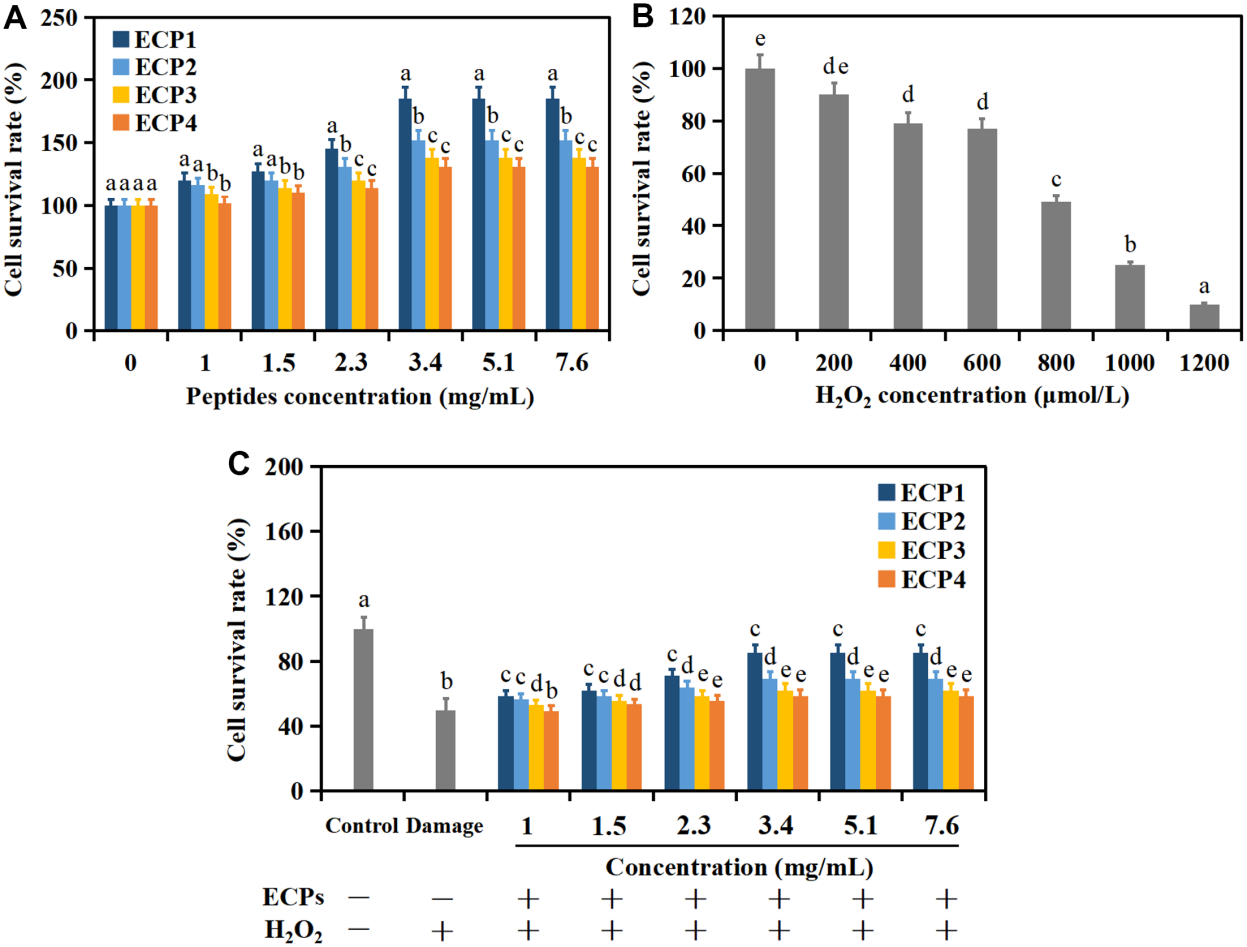
The survival rates of HepG2 cells treated with ECPs (A), H_2_O_2_ (B), both ECPs and H_2_O_2_ (C). Values are mean of triplicate samples.

H_2_O_2_ is an important reactive oxygen species, which is stable and extremely easy to penetrate cell membrane. As an oxidant to induce the establishment of cell oxidative damage model, H_2_O_2_ is widely used in scientific research [23]. The survival rates of cells cultured with ECPs for 24 h were first investigated (Fig. 2A). Obviously, the survival rates went up with the rise of peptides concentration, but remained basically unaltered with the rise of peptides concentration at 3.4-7.6 mg ml^–1^. In particular, ECP1 presented the most obvious effect on improving cell survival rate in comparison with ECP2, ECP3 and ECP4.

The survival rates of oxidative injured cells treated with H_2_O_2_ (0-1200 μmol l^–1^, 2 h) were also measured (Fig. 2B). Cell survival rates remarkable went down with the rise of H_2_O_2_ concentration, and was closest to 50% at the concentration of 800 μmol l^–1^. So, the oxidative injured cell model was established.

Besides, survival rates of cells precultured with ECPs for 24 h, following by treating with H_2_O_2_ for 2 h were investigated (Fig. 2C). Compared with the oxidative injured group, survival rates of cells pretreated with ECPs improved. Particularly, ECP1 still showed the best protective effect on increasing survival rate in comparison with ECP2, ECP3 and ECP4. These results indicated that peptides from *C. militaris* cultivated with tussah pupa could protect cells from oxidative injured, and that the low molecular weight peptides (ECP1) were more effective than other molecular weight peptides (ECP2, ECP3 and ECP4) in promoting HepG2 cells proliferation. Hu *et al*. found that peptides of grass carp scale gelatin also improved survival rates of HepG2 cells [3].

### Apoptosis Rate

**Fig. 3:**
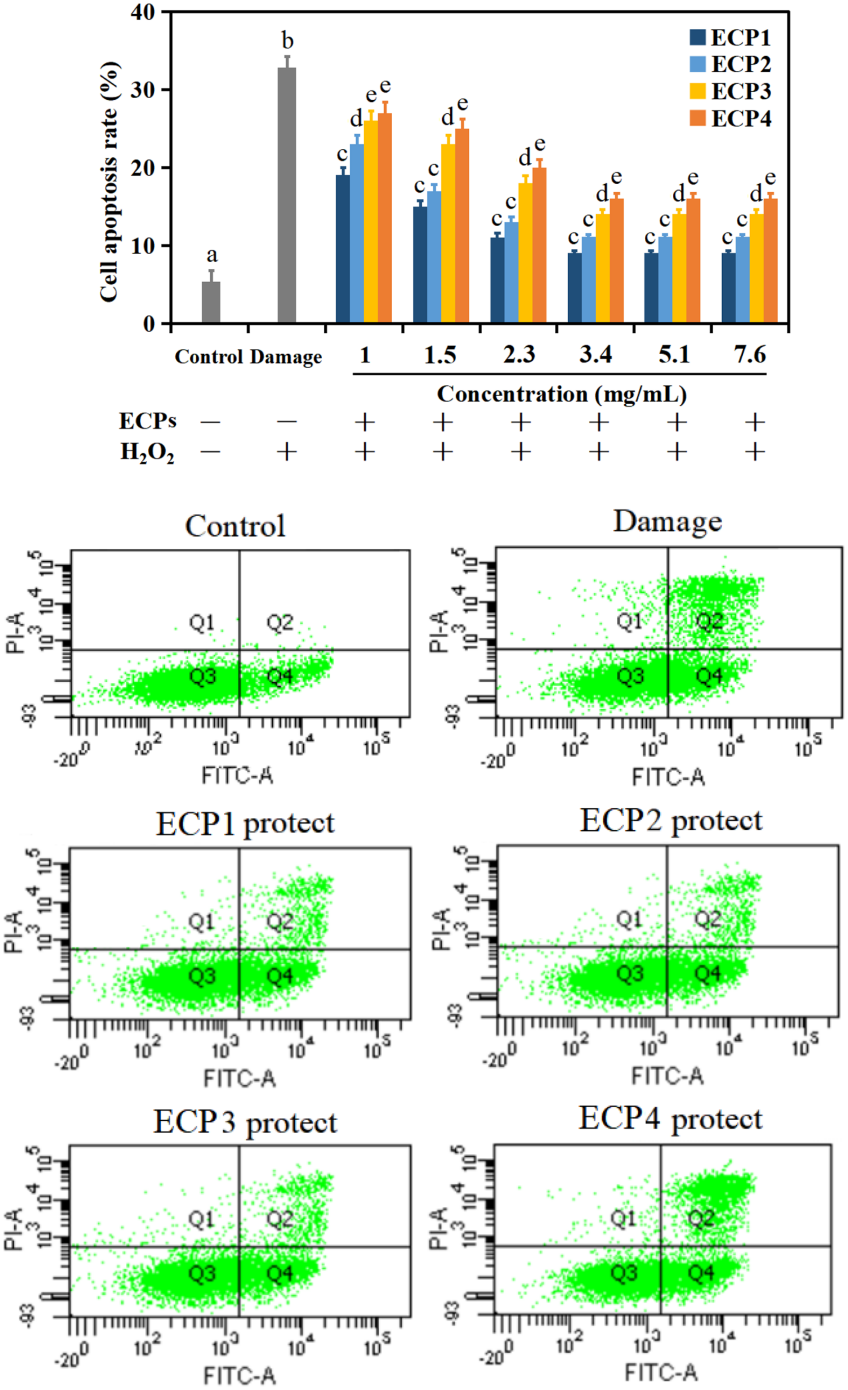
The apoptosis rates of HepG2 cells. Values are mean of triplicate samples.

Flow cytometry (FCM) can quickly and accurately detect the proportion and degree of cell apoptosis, which is an important biological technology to study apoptosis FITC-A, located on the X-axis, is a fluorescent probe commonly used in biological studies that binds specifically to phosphatidylserine (PS) with high affinity. In the early stage of apoptosis, the phosphatidylserine (PS), which is only distributed inside the lipid bilayer of normal cell membranes, is turned laterally, and Annexin V labeled fluorescein is used as a fluorescent probe, coupled with nucleic acid dyes such as PI located on the y axis, to accurately distinguish between normal cells, early apoptotic cells and late apoptotic cells [3, 22]. Fig. 3 showed that apoptosis rate in control group was 5.2%. When cells treated with H_2_O_2_ (800 μmol l^–1^, 2 h), cell apoptosis rate was up to 33 %. However, apoptosis rates of cells pretreated with ECPs were observably went down. ECP1 presented excellent effect in inhibiting the rise of apoptosis rate compared to ECP2, ECP3 and ECP4. In addition, the inhibitory effect on apoptosis of HepG2 cells was more obvious with the increase of peptides concentration at 1-3.4 mg ml^–1^, but kept almost steady with the continuous increase of concentration at 3.4-7.6 mg ml^–1^. The above results suggested that peptides from *C. militaris* cultivated with tussah pupa could protect cells from oxidative injured. Also, the low molecular weight peptides (ECP1, <3 kDa) showed the most remarkable protective effect.

### ROS Level and MDA Content

**Fig. 4:**
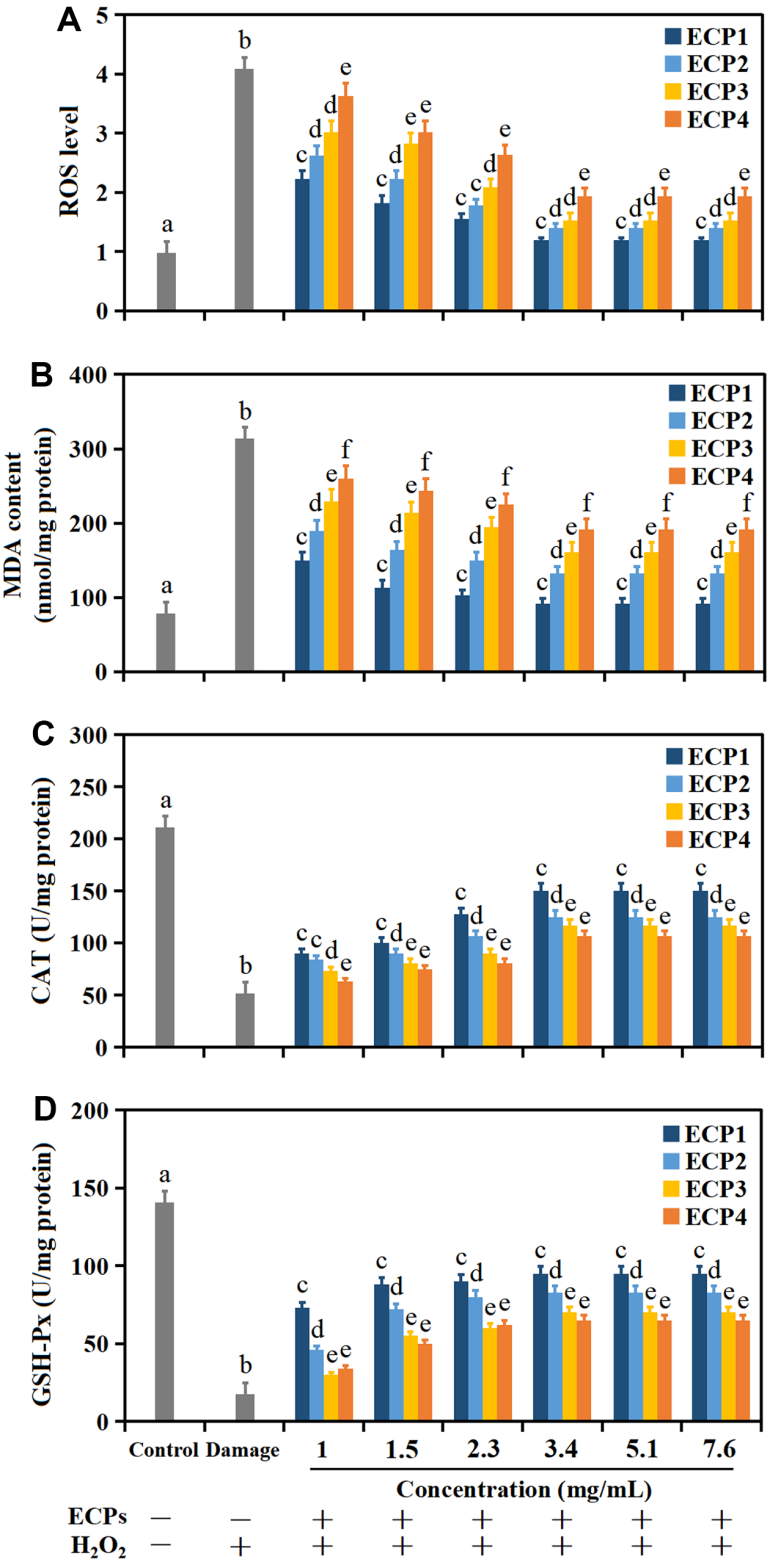
ROS level (A), MDA content (B), CAT (C) and GSH-Px (D) activities of HepG2 cells. Values are mean of triplicate samples.

ROS level and MDA content can directly or indirectly show the level of oxidative damage [24, 25]. As shown in Fig. 4, ROS level and MDA content observably increased (*p* < 0.05) in comparison with the control. Pretreatment with ECPs inhibited the increase of ROS level and MDA content, but the effects kept basically unchanged with the increase of peptides concentration at 3.4-7.6 mg ml^–1^. In particular, ECP1 presented the most obvious effect on restraining the increase of ROS level and MDA content in comparison with ECP2, ECP3 and ECP4. The results showed that ECPs treatment could inhibit lipid peroxidation of cell membrane to a certain extent, thus protecting cell membrane from free radical damage. Li *et al*. considered that peptides from maggot could significantly reduce the ROS level of IPEC-J2 cells damaged by oxidation [24].

### CAT and GSH-Px Activities

Antioxidants are important substances in the resistance to oxidative damage, including enzyme antioxidant system (CAT, SOD, GPX) and non-enzyme antioxidant system (GSH-Px, carotene, Vitamin E) [26]. The CAT and GSH-Px activities in HepG2 cells were detected to evaluate the protective effect of peptides from *C. militaris* cultivated with tussah pupa. As shown in Fig. 4C and 4D, the CAT and GSH-Px activities in H_2_O_2_-treated HepG2 cells observably decreased (*p* < 0.05) in comparing to the untreated group, and the decrements were 76.2% and 85.7%, respectively. Pretreatment with ECPs improved ROS level and MDA content of oxidative injured cells, and the low molecular weight peptides (ECP1, <3 kDa) likewise showed the most remarkable protective effect. Low molecular weight peptides can be directly absorbed and used by the body, and this mechanism determines the efficient utilization and conversion rate of low molecular weight peptides. The reason may be that low molecular weight peptides are simple in structure and can pass through the cellular lipid membrane more efficiently than high molecular weight peptides [21]. Besides, low molecular weight peptides may contain special antioxidant amino acid residues capable of interacting with proteins or nucleic acids, which associating with the antioxidant defense system or ROS dependent signaling pathways [2]. For example, lupin antioxidant peptides increased the expression level of antioxidant genes in the cell and further enhanced the activities of antioxidant enzymes. At the same time, antioxidant peptides also induced the up-regulation of a series of endogenous cytoprotective genes by activating the Nrf2-ARE pathway, thereby strengthening the antioxidant ability of the intracellular antioxidant system and inhibiting oxidative damage [27].

The above results indicated that low molecular weight peptides (<3 kDa) from *Cordyceps militaris* cultivated with tussah pupa had the best antioxidant capacity and protective effect on oxidative injured HepG2 cells. So, ECP1 was selected for the following characterization study.

### The Micromorphology of NCP1 and ECP1

**Fig. 5:**
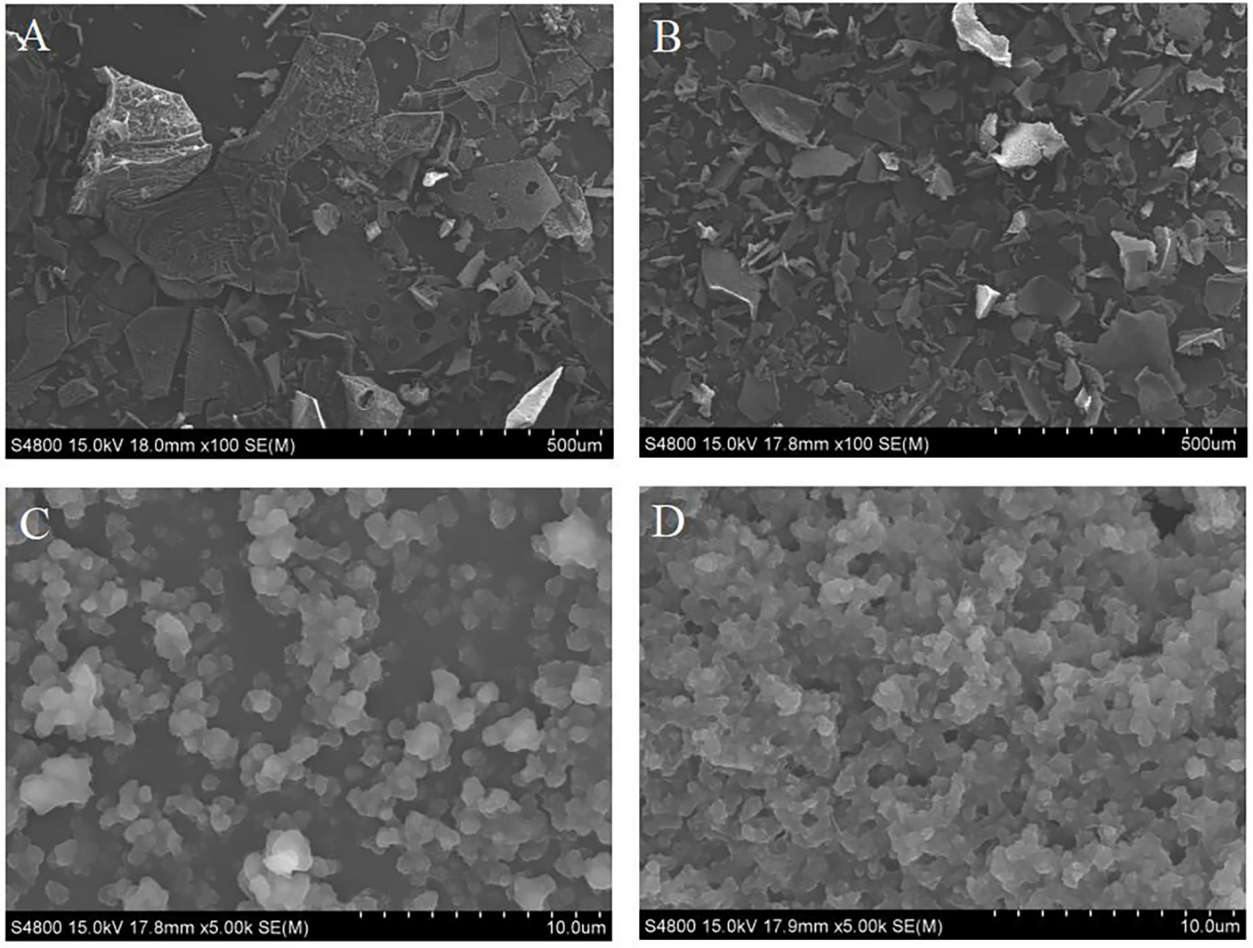
SEM images of NCP1 (A and C) and ECP1 (B and D).

The differences in micromorphology between NCP1 and ECP1 were characterized by SEM. NCP1 presented irregular massive crystals with rough surfaces (Fig. 5A), while ECP1 showed uniform flake-like crystals with smooth surfaces (Fig. 5B). Magnification by SEM showed that the surfaces of ECP1 appeared loose porous spongy structures (Fig. 5D) compared with that of NCP1 (Fig. 5C), which indicated that enzymatic hydrolysis destroyed the complete structures of NCP1. Therefore, it can be concluded that the change of micromorphology may be one of the reasons for the significant enhancement of antioxidant capacity of ECP1.

### DSC

**Fig. 6:**
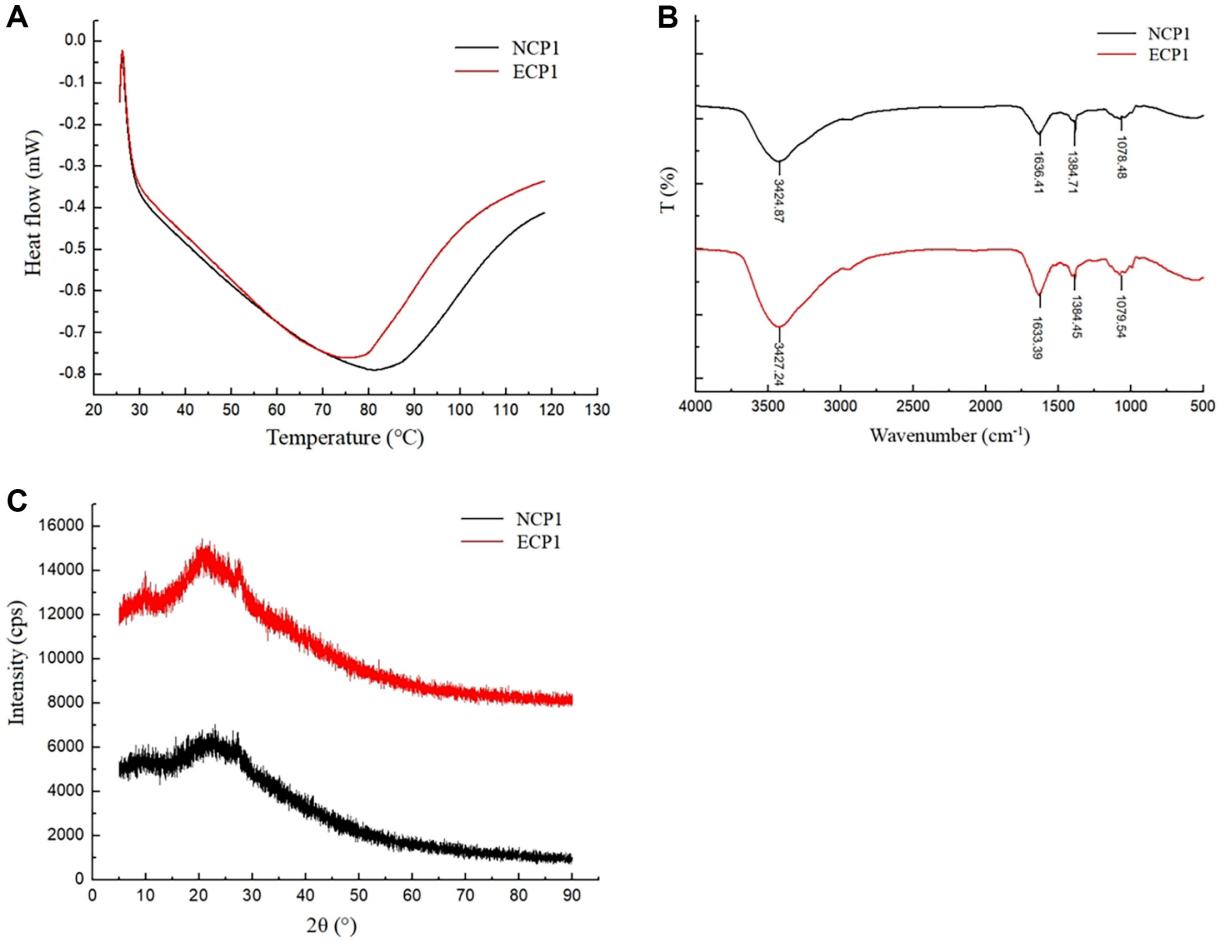
DSC curves (A), FT-IR spectra (B) and XRD patterns (C) of NCP1 and ECP1.

DSC curve can show the degree of denaturation and structural changes of the peptide or protein [4]. The DSC curves of NCP1 and ECP1 were presented in Fig. 6A. In the curve of NCP1, a broad endothermic peak at 82°C was observed. However, the DSC curve of ECP1 had a relatively narrow endothermic peak at 74°C, which was observably weakened as compared with the NCP1. The results indicated that the physical form of NCP1 was changed by enzymatic hydrolysis.

### FT-IR

It was seen that almost identical absorption peaks were observed in the spectra of NCP1 and ECP1 (Fig. 6B), which demonstrated that no new chemical bonds had been produced in ECP1 as compared with the NCP1. The stretching vibrations peak of N-H and O-H was observed at 3424.87 cm^–1^ in NCP1, while the peak in ECP1 was weaker and shifted to 3427.24 cm^–1^. Meanwhile, the stretching vibrations peaks at 1384.71 cm^–1^ and 1078.48 cm^–1^ corresponding to C-N and C-O were slightly shifted. Besides, the N-H stretching vibrations peak shifted from 1636.41 cm^–1^ to 1633.39 cm^–1^. The results showed that the complete helical structure of the NCP1 was destroyed, exposing the tightly packed active group, which was more susceptible to electron capture by free radicals. All phenomena above further demonstrated that ECP1 had better antioxidant capacity than NCP1, which was consistent with our previous results [28].

### XRD

The XRD spectrum showed the differences of crystal structure between NCP1 and ECP1 (Fig. 6C). A total of three relatively obvious diffraction peaks were observed in the XRD spectrum of NCP1 and ECP1. The three diffraction peaks in spectrum of ECP1 significantly enhanced as compared with that of NCP1. The results showed that enzymatic hydrolysis generated the rearrangement and aggregation of macromolecular proteins, which changed the crystal structure.

### AAC

The biological activity of peptides is related to amino acid composition and amino acid sequence. It has been reported that hydrophobic amino acids due to their strong hydrophobicity can increase the interaction with lipids, thus enhancing the ability of peptides to inhibit lipid oxidation. Acidic amino acids can provide excess electrons, which contributes to enhance its antioxidant activity [29]. Comparative analysis of amino acid content in NCP1 and ECP1 was shown in Table 1. Compared with NCP1, most of hydrophobic amino acids (threonine, alanine, valine, methionine, isoleucine, phenylalanine and proline) and acidic amino acids (aspartate and glutamate) contents in ECP1 were observably improved (*p* < 0.05) by enzymatic hydrolysis. The results showed that the inhibition of lipid oxidation and antioxidant capacity of ECP1 were enhanced, which also indicated the antioxidant activity was closely related to the ACC.

## Conclusion

Pretreated with ECP1 (<3 kDa) showed the strongest antioxidant capacity comparing to the other molecular weight peptides, and the antioxidant effect was positively correlated with the concentration. ECPs treatment significantly enhanced the survival rates, reduced the apoptosis rates, decreased the ROS level and MDA content, and increased CAT and GSH-Px activities of oxidative injured HepG2 cells. Obviously, ECP1 had the best protective effect on oxidative injured HepG2 cells. Moreover, pretreated with ECP1 suppressed the autophagy of oxidative injured HepG2 cells, which was consistent with the results of apoptosis rates. In addition, the DSC, FT-IR and XRD results indicated that the structure of NCP1 were changed by enzymatic hydrolysis treatment. Most of hydrophobic and acidic amino acids contents in ECP1 were also observably improved (*p* < 0.05) by enzymatic hydrolysis. So, we concluded that low molecular weight peptides (ECP1, <3 kDa) had more potential research value for the developing functional foods or antioxidant cosmetics. Deeper researches are required to explore the detailed analysis of amino acid sequences or 3D structures of ECP1, which will contribute to the development of functional foods or pharmaceuticals.

## Figures and Tables

**Table 1:** Comparative analysis of amino acid content in NCP1 and ECP1.

Name of amino acid	NCP1 (mg g^–1^)	ECP1 (mg g^–1^)
Aspartate	18.66 ± 0.03 a	20.26 ± 0.09 b
Threonine	11.02 ± 0.12 a	15.80 ± 0.06 b
Serine	10.91 ± 0.09 a	10.77 ± 0.15 a
Glutamate	22.41 ± 0.24 a	25.91 ± 0.27 b
Glycine	14.41 ± 0.16 a	14.98 ± 0.07 a
Alanine	11.64 ± 0.22 a	13.63 ± 0.32 b
Cystine	2.73 ± 0.05 a	2.57 ± 0.05 a
Valine	14.72 ± 0.10 a	16.67 ± 0.09 b
Methionine	13.66 ± 0.13 a	16.49 ± 0.14 b
Isoleucine	4.28 ± 0.11 a	6.20 ± 0.05 b
Leucine	8.31 ± 0.06 a	8.99 ± 0.02 a
Tyrosine	14.49 ± 0.08 a	14.39 ± 0.18 a
Phenylalanine	8.46 ± 0.21 a	10.43 ± 0.06 b
Lysine	15.29 ± 0.20 a	15.77 ± 0.12 a
Histidine	6.37 ± 0.09 a	6.42 ± 0.11 a
Arginine	14.10 ± 0.32 a	14.85 ± 0.25 a
Proline	10.46 ± 0.05 a	12.43 ± 0.07 b

All means in the same row followed by different letters are significantly different (*p* < 0.05).
